# Protocol for morphogen-guided differentiation of brain cell types using human induced pluripotent stem cells

**DOI:** 10.1016/j.xpro.2025.104339

**Published:** 2026-01-16

**Authors:** Lu Qian, Juao-Guilherme Rosa, Julia TCW

**Affiliations:** 1 Department of Pharmacology, Physiology & Biophysics, Boston University Chobanian & Avedisian School of Medicine, Boston, MA 02118, USA; 2 Neuroscience Program, Boston University, Boston, MA 02215, USA

**Keywords:** Cell culture, Neuroscience, Stem cells, Cell differentiation

## Abstract

Modeling neurological disorders is challenging due to differing functional genomics and phenotypes among species. Here, we present a protocol for morphogen-guided differentiation of human induced pluripotent stem cells (hiPSCs) into microglia, astrocytes, and mixed cortical cultures (MCCs) for studying human brain disorders. We describe steps for enhancing microglial production using hypoxia and implementing quality-control measures for astrocyte and MCC differentiations. We detail knockout serum replacement procedures for serum-free astrocytes. This protocol enables cell-type-specific investigation of disease mechanisms and drug screening.

## Highlights

• Guidance for morphogen- and small-molecule differentiation of human iPSC neural cultures
• Steps for hypoxia-facilitated differentiation of hematopoietic progenitors at high yield
• Instructions for serum-free astrocyte differentiation
• Procedures for troubleshooting neural differentiation with contaminant cell types

## Abstract (editor highlights)

Publisher’s note: Undertaking any experimental protocol requires adherence to local institutional guidelines for laboratory safety and ethics.

## Graphical abstract

**Figure:**
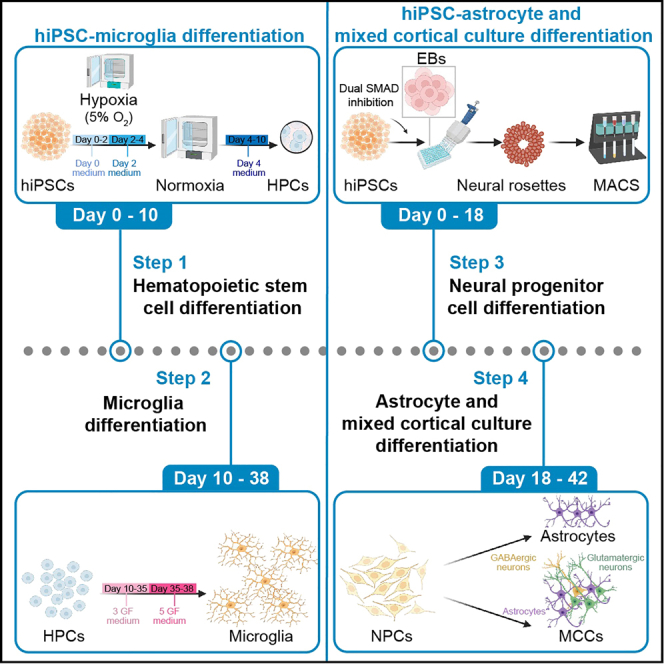

## Teaser

Modeling neurological disorders is challenging due to differing functional genomics and phenotypes among species. Here, we present a protocol for morphogen-guided differentiation of human induced pluripotent stem cells (hiPSCs) into microglia, astrocytes, and mixed cortical cultures (MCCs) for studying human brain disorders. We describe steps for enhancing microglial production using hypoxia and implementing quality-control measures for astrocyte and MCC differentiation. We detail knockout serum replacement procedures for serum-free astrocytes. This protocol enables cell-type-specific investigation of disease mechanisms and drug screening.

## Before you begin

This protocol outlines the differentiation of hematopoietic progenitor cells (HPCs), followed by microglia and neural progenitor cells (NPCs), followed by astrocytes and mixed cortical cultures from human induced pluripotent cells (hiPSC). We also describe approaches for cell identity validation via fluorescence-activated cell sorting (FACS), magnetic-activated cell sorting (MACS), and immunocytochemistry. Before initiating any differentiation, all hiPSCs must be quality-controlled for mycoplasma negative, karyotype normal, and pluripotency. Once the hiPSC lines are established and stored in cryopreserved stocks, this differentiation protocol can proceed. Reconstitution of small molecules and growth factors in aliquots and preparation of cell culture media are required before starting the protocol. It is recommended to plan out the timeline and usage of supplemented media across stages of differentiation to avoid expiration of reagents and growth factors.

### Innovation

The development of the cerebral cortex is complicated by cell proliferation, neuronal migration, and cortical organization. This protocol mimics key aspects of embryonic neurogenesis and gliogenesis, highlighting the use of directed differentiation to replicate essential signaling cues during cortical development. Existing methods for differentiating hiPSC-derived microglia, including induced and directed differentiation using transcription factors or a commercial kit,^1^^,^^2^ face limitations in low scalability, a lack of specific myeloid and microglial markers, and low reproducibility. To address these challenges, this protocol uses hypoxia (5% O_2_) to generate mature microglia, achieving approximately an 8-fold increase in cell number from the input hiPSCs. The microglia demonstrate competent phagocytosis and enhanced cholesterol efflux in the presence of 2% high-density lipoprotein.^3^ Furthermore, the protocol introduces a new method for efficiently differentiating mature, functional astrocytes and mixed cortical cultures (MCCs) from NPCs. We described streamlined hiPSC-NPC differentiation via dual SMAD inhibition, ensuring experimental reproducibility and cell purity by MACS and performing quality control through co-expression analysis of NPC markers such as SOX2, PAX6, and NESTIN. For astrocyte differentiation, we present a scalable platform utilizing astrocyte medium, validated by healthy spontaneous calcium spikes and phagocytosis of myelin bioparticles.^3^^,^^4^ We also describe the use of KnockOut Serum Replacement (KOSR) to generate homeostatically equivalent astrocytes as in the presence of serum without stimulating cellular metabolism.^3^ Lastly, the NPC quality control by MACS maintains cell-type consistency of the 6-week-cultured MCCs, allowing us to study genotype effects even in the face of multiple cell types in the culture.

### Preparation for human induced pluripotent stem cell culture

**Timing: 1 day**

1. Coat a tissue culture-treated 6-well plate with growth factor-reduced Matrigel.
  a. Pre-rinse pipette with ice-cold DMEM.
  b. Dilute 192 μL of a frozen Matrigel (stock concentration range 8 - 11mg/mL) aliquot in 48 mL of ice-cold DMEM; gently pipette up and down to mix well.
  c. Add 2 mL of the reconstituted Matrigel solution per 6-well and distribute evenly.
  d. Incubate at 37°C and 5% CO_2_ for 1 h.

***Note:*** If not used immediately, the Matrigel-coated plates can be stored in the incubator for up to 1 week or parafilmed to avoid evaporation and stored at 4°C for 2 weeks to 1 month.

2. Make a complete mTeSR Plus medium.
  a. Thaw 100 mL mTeSR Plus supplement (5x) and mix with 400 mL mTeSR Plus basal medium for a final 1x concentration.
  b. Supplement the complete mTeSR Plus medium with 1x Antibiotic-Antimycotic.

***Note:*** The complete mTeSR Plus medium can be stored at 4°C for up to 2 weeks. If not used immediately, the aliquoted medium can be stored at −20°C for up to 6 months.

### Reconstitution and storage of small molecules and growth factors

**Timing: 2 h**

3. Prepare aliquots of stock solutions to reduce frequent freeze-and-thaw cycles. See Tables 1, 2, 3, and 4 for storage and working concentration.

**Table 1:** Storage and working concentrations of reagents to differentiate HPC

Reagent	Storage concentration	Working concentration	Storage conditions
Monothioglycerol (MTG)	400 mM	400 μM	4°C
Polyvinyl Alcohol (PVA)	10 mg/mL	10 μg/mL	
Insulin	10 mg/mL	20 μg/mL	
Ascorbic Acid	36 mg/mL	64 μg/mL	−20°C
FGF2	100 μg/mL	50 ng/mL	
BMP4	50 μg/mL	50 ng/mL	
Activin A	12.5 μg/mL	12.5 ng/mL	
LiCl	2 M	2 mM	
Thiazovivin (Tzv)	10 mM	10 μM	
VEGF	50 μg/mL	50 ng/mL	
TPO	50 μg/mL	50 ng/mL	
SCF	100 μg/mL	10 ng/mL	
IL-6	100 μg/mL	50 ng/mL	
IL-3	100 μg/mL	10 ng/mL

**Table 2:** Storage and working concentrations of reagents to differentiate microglia

Reagent	Storage concentration	Working concentration	Storage conditions
Monothioglycerol (MTG)	400 mM	200 μM	4°C
Insulin	10 mg/mL	5 μg/mL	
TGFB	50 μg/mL	50 ng/mL	−20°C
IL-34	100 μg/mL	100 ng/mL	
MCSF	25 μg/mL	25 ng/mL	
CX3CL1	100 μg/mL	100 ng/mL	
CD200	100 μg/mL	100 ng/mL

**Table 3:** Storage and working concentrations of reagents to differentiate embryonic bodies (EB) and NPC

Reagent	Storage concentration	Working concentration	Storage conditions
LDN193189	100 μM	0.1 μM	−20°C
SB431542	10 mM	10 μM	
FGF2	20 μg/mL	20 ng/mL

**Table 4:** Storage and working concentrations of reagents to differentiate MCC

Reagent	Storage concentration	Working concentration	Storage conditions
BDNF	20 μg/mL	20 ng/mL	−20°C
GDNF	20 μg/mL	20 ng/mL	
Dibutyryl Cyclic AMP Sodium Salt (cAMP)	100 mg/mL	250 μg/mL	
L-Ascorbic Acid	200 mM	200 μM	
Laminin	1 mg/mL	1 μg/mL

***Note:*** Reconstituted aliquots of monothioglycerol (MTG) and polyvinyl Alcohol (PVA) can be stored at 4°C for up to 1 month; product shelf-life is applied to insulin storage at 4°C. Reconstituted aliquots of growth factors and cytokines can be stored at −20°C for up to 6–8 months.

### Preparation of HPC differentiation medium

**Timing: 30 min**

4. Prepare HPC basal medium using Table 5.
  a. Combine an equal volume of IMDM and F12 medium.
  b. Add 10 μg/mL PVA, 1% (vol/vol) Antibiotic-Antimycotic (100x), 1% (vol/vol) GlutaMax (100x), 1% (vol/vol) MEM NEAA (100x), 1% (vol/vol) CD lipid concentrate, 64 μg/mL ascorbic acid, 2% (vol/vol) ITS-X, 20 μg/mL insulin and 400 μM MTG to the IMDM/F12 combined medium.

**Table 5:** Composition of HPC basal medium

Medium	Reagent	Volume for 100 mL	Dilution
HPC Basal Medium	IMDM	46.8 mL	50% v/v
	F12	46.8 mL	50% v/v
	Antibiotic-Antimycotic	1 mL	1:100
	PVA	100 μL	1:1,000
	GlutaMAX	1 mL	1:100
	NEAA	1 mL	1:100
	CD Lipid Concentrate	1 mL	1:100
	Ascorbic Acid	178 μL	1:562
	ITS-X	2 mL	1:50
	MTG	100 μL	1:1,000
	Insulin	200 μL	1:500
5. Prepare HPC d0 - 2 medium using Table 6.
  a. Add 50 ng/mL FGF2, 50 ng/mL BMP4, 12.5 ng/mL activin A, 2mM LiCl, and 10 μM of thiazovivin (Tzv) to the HPC basal medium.

**Table 6:** Composition of HPC differentiation medium factors from Day 0 to 2

Differentiation stage	Reagent	Volume for 100 mL	Dilution
Day 0	FGF2	50 μL	1:2,000
	BMP4	100 μL	1:1,000
	Activin A	100 μL	1:1,000
	LiCl	100 μL	1:1,000
	Thiazovivin (Tzv)	100 μL	1:1,000
6. Prepare HPC d2 - 4 medium using Table 7.
  a. Add 50 ng/mL FGF2, 50 ng/mL VEGF to the HPC basal medium.

**Table 7:** Composition of HPC differentiation medium factors from Day 2 to 4

Differentiation stage	Reagent	Volume for 100 mL	Dilution
Day 2 - 4	FGF2	50 μL	1:2,000
	VEGF	100 μL	1:1,000
7. Prepare HPC d4 - 10 medium using Table 8.
  a. Add 50 ng/mL FGF2, 50 ng/mL VEGF, 50 ng/mL TPO, 10 ng/mL SCF, 50 ng/mL IL-6, and 10 ng/mL IL-3 to the HPC basal medium.

**Table 8:** Composition of HPC differentiation medium from Day 4 to 10

Differentiation stage	Reagent	Volume for 100 mL	Dilution
Day 4 - 10	FGF2	50 μL	1:2,000
	VEGF	100 μL	1:1,000
	TPO	100 μL	1:1,000
	SCF	10 μL	1:10,000
	IL-6	50 μL	1:2,000
	IL-3	10 μL	1:10,000

***Note:*** The medium can be stored for up to 2 weeks at 4°C.

**CRITICAL:** HPC differentiation from Day 0 to Day 3 takes place under hypoxia. Pre-equilibrate the media in the multi-gas hypoxia incubator (5% O_2_ and 5% CO_2_ setting) for 1 h at 37°C before use.

### Preparation of microglia differentiation medium

**Timing: 30 min**

8. Prepare microglia medium according to differentiation stages. Compose the microglia basal medium using Table 9.
  a. Add 1% (vol/vol) Antibiotic-Antimycotic (100x), 1% (vol/vol) GlutaMax (100x), 1% (vol/vol) MEM NEAA (100x), 2% (vol/vol) ITS-G, 2% (vol/vol) B27, 0.5% (vol/vol) N2 supplement, 200 μM MTG, and 5 μg/mL insulin to the phenol-free DMEM/F12.

**Table 9:** Composition of microglia basal medium

Medium	Reagent	Volume for 100 mL	Dilution
Microglia Basal Medium	Phenol-free DMEM/F12 1:1	92.4 mL	1x
	GlutaMax	1 mL	1:100
	NEAA	1 mL	1:100
	ITS-G	2 mL	1:50
	B27 with Vitamin A	2 mL	1:50
	N2	0.5 mL	1:200
	MTG	50 μL	1:2,000
	Insulin	50 μL	1:2,000
	Antibiotic-Antimycotic	1 mL	1: 100
9. Prepare microglia medium Day 0–25 (3GF) medium using Table 10.
  a. Add 100 ng/mL IL-34, 25 ng/mL MCSF, and 50 ng/mL TGFβ to the microglia basal medium.

**Table 10:** Composition of microglia differentiation factors from Day 0 to Day 25 (3 GF) and from Day 25 to Day 28 (5 GF)

Differentiation stage	Reagent	Volume for 100 mL	Dilution
Day 0 - 25 (3 GF)	TGFB	100 μL	1:1,000
	IL-34	100 μL	1:1,000
	M-CSF	100 μL	1:1,000
Day 25 - 28 (5 GF)	CX3CL1	100 μL	1:1,000
	CD200	100 μL	1:1,000
10. Prepare microglia medium Day 25–28 (5GF) medium using Table 10.
  a. Add 100 ng/mL IL-34, 25 ng/mL MCSF, 50 ng/mL TGFβ, 100 ng/mL CX3CL1, and 100 ng/mL CD200 to the microglia basal medium.

***Note:*** The medium can be stored for up to 2 weeks at 4°C.

### Preparation of embryonic body (EB) differentiation medium

**Timing: 30 min**

11. Prepare the basal differentiation medium using Table 11.
  a. Add 1% (vol/vol) Antibiotic-Antimycotic (100x), 1% (vol/vol) N2 supplement, and 2% (vol/vol) B27 without Vitamin A to the DMEM/F12 with GlutaMax medium.

**Table 11:** Composition of EB differentiation medium

Reagent	Storage concentration	Working concentration	Volume for 100 mL	Dilution
DMEM/F12 with GlutaMax	1x	1x	95.8 mL	1x
N2 Supplement	100x	1x	1 mL	1:100
B27 without Vitamin A	50x	1x	2 mL	1:50
Antibiotic-Antimycotic	100x	1x	1 mL	1:100
LDN193189	100 μM	0.1 μM	100 μL	1:1,000
SB431542	10 mM	10 μM	100 μL	1:1,000
12. Add 0.1 μM LDN193189 and 10 μM SB431542 to the basal differentiation medium.

***Note:*** The medium can be stored for up to 2 weeks at 4°C.

### Preparation of NPC differentiation medium

**Timing: 30 min**

13. Prepare the basal differentiation medium using Table 12.
  a. Add 1% (vol/vol) Antibiotic-Antimycotic (100x), 1% (vol/vol) N2 supplement, and 2% (vol/vol) B27 without Vitamin A to the DMEM/F12 with GlutaMax medium.

**Table 12:** Composition of NPC differentiation medium

Reagent	Storage concentration	Working concentration	Volume for 100 mL	Dilution
DMEM/F12 with GlutaMax	1x	1x	95.9 mL	1x
N2 Supplement	100x	1x	1 mL	1:100
B27 without Vitamin A	50x	1x	2 mL	1:50
Antibiotic-Antimycotic	100x	1x	1 mL	1:100
FGF2	20 μg/mL	20 ng/mL	100 μL	1:1,000
14. Add 20 ng/ml FGF2 to the basal differentiation medium.

***Note:*** The medium can be stored for up to 2 weeks at 4°C.

### Media for astrocyte differentiation

**Timing: 30 min**

15. Prepare the astrocyte differentiation medium using Table 13.
  a. Add 1% (vol/vol) AGS, 1% (vol/vol) P/S, and 2% (vol/vol) FBS to the astrocyte basal medium per manufacturer’s instruction.

**Table 13:** Composition of astrocyte differentiation medium

Reagent	Storage concentration	Working concentration	Volume for 100 mL	Dilution
Astrocyte Basal Medium	1x	1x	96 mL	1x
Fetal Bovine Serum (FBS)	50x	1x	2 mL	1:50
Astrocyte Growth Factors (AGS)	100x	1x	1 mL	1:100
Penicillin/Streptomycin (P/S)	100x	1x	1 mL	1:100

***Note:*** Aliquot the completely supplemented astrocyte differentiation medium into smaller volumes and store under −20°C for extended storage. Do not leave the supplemented medium over 1 month at 4°C.

### Media for mixed cortical culture (MCC) differentiation

**Timing: 30 min**

16. Prepare MCC differentiation medium using Table 14.
  a. Add 1% (vol/vol) Antibiotic-Antimycotic (100x), 1% (vol/vol) N2 supplement, 2% (vol/vol) B27 without Vitamin A to the BrainPhys medium.
  b. Add 20 ng/mL BDNF, 20 ng/mL GDNF, 250 μg/mL cAMP, 200 μM L-Ascorbic Acid, and 1 μg/ml of Laminin to the supplemented BrainPhys medium.

**Table 14:** Composition of MCC differentiation medium

Reagent	Storage concentration	Working concentration	Volume for 100 mL	Dilution
BrainPhys	1x	1x	95.35 mL	1x
N2 Supplement	100x	1x	1 mL	1:100
B27 without vitamin A	50x	1x	2 mL	1:50
Antibiotic-Antimycotic	100x	1x	1 mL	1:100
BDNF	20 μg/mL	20 ng/mL	100 μL	1:1,000
GDNF	20 μg/mL	20 ng/mL	100 μL	1:1,000
Dibutyryl Cyclic AMP Sodium Salt (cAMP)	100 mg/ml	250 μg/mL	250 μL	1:400
L-Ascorbic Acid	200 mM	200 μM	100 μL	1:1,000
Laminin	1 mg/ml	1 μg/ml	100 μL	1:1,000

***Note:*** Aliquot the MCC differentiation medium from 1a into smaller volumes and store under −20°C for extended storage. MCC differentiation medium supplemented with growth factors from 1b can be stored for up to 2 weeks at 4°C.

## Key resources table

**Table:** 

REAGENT or RESOURCE	SOURCE	IDENTIFIER
**Experimental models: Cell lines**		

CRISPR-edited isogenic hiPSC lines generated by the TCW Lab	TCW et al.^1^	TCW1E33-1C6, TCW1E33-1C8, TCW1E33-1F1, TCW1E44-2C2, TCW1E44-2E3, TCW1E44-1D1, TCW1EKO-1A12, TCW1EKO-1B2, TCW1EKO-1B12, TCW2E33-2E3, TCW2E33-3A5, TCW2E33-3D11, TCW2E44-4B1, TCW2E44-4B4, TCW2E44-4B12, TCW2EKO-3A5, TCW2EKO-3A12, TCW2EKO-3D10, TCW3E33-RC1B TCW3E33-RC1H, TCW3E33-RC1I, TCW3E44-CD1D, TCW3E44-CD1F, TCW4E33-MC2C, TCW4E33-MC2E, TCW4E33-MC2F, TCW4E44-RC2C, TCW4E44-RC2G, TCW4E44-RC2I
KOLF2.1J	The Jackson Laboratory	JIPSC001000

**Critical commercial assays**		

Neural Crest Stem Cell MicroBeads	Miltenyi Biotec	Cat# 130-097-127
Indirect CD133 MicroBeads	Miltenyi Biotec	Cat# 130-091-895

**Antibodies**		

SOX2 (1:100)	Cell Signaling	Cat# 3579 S, RRID:AB_2195767
PAX6 (1:100)	Abcam	Cat# ab5790, RRID:AB_305110
FOXP2 (1:100)	Abcam	Cat# ab16046, RRID:AB_2107107
NESTIN (1:100)	Abcam	Cat# ab22035, RRID:AB_446723
CD43 (clone CD43-10G7) (1:50)	BioLegend	Cat# 343206, RRID: AB_2194072
CD45 (HI30) (1:50)	Tonbo Bioscience	Cat# 25-0459, RRID:AB_2621631
CS235a (Glycophorin A) (clone HI264) (1:50)	BioLegend	Cat# 349112, RRID: AB_2562708
CD41 (clone HIP8) (1:50)	BioLegend	Cat# 303706, RRID: AB_314376
S100β (1:1000)	Sigma	Cat# S2532, RRID:AB_477499
Vimentin (1:500)	Cell Signaling	Cat# R28#3932
CX3CR1 (1:500)	BioRad	Cat# AHP1589, RRID:AB_2087421
IBA1 (1:300)	Sigma	Cat# MABN92, RRID:AB_10917271
TREM2 (1:100)	R&D	Cat# AF1828, RRID:AB_2208689
P2RY12 (1:1000)	Sigma	Cat# HPA014518, RRID:AB_2669027
PU.1 (1:100)	Cell Signaling	Cat# 2266, RRID:AB_10692379
GFAP (1:200)	Millipore	Cat# MAB3402, RRID:AB_94844
TMEM119 (1:100)	Abcam	Cat# ab185333
HCS LipidTOX™ Deep Red neutral lipid stain (1:200)	Invitrogen	Cat# H34475

**Chemical, peptides and recombinant proteins**		

LDN193189	Stemgent	Cat# 04-0074
SB431542	Stemgent	Cat# 04-0010
FGF2	R&D	Cat# 233-FB
BMP4	R&D	Cat# 314-BP
Activin A	R&D	Cat# 338-AC
VEGF	R&D	Cat# 293-VE
Poly-vinyl-Alcohol (PVA)	Sigma	Cat# P8136
Thiazovivin (Tzv)	Millipore	Cat# 420220
Human Insulin	Sigma	Cat# I2643
TPO	R&D	Cat# 288-TP
IL-6	R&D	Cat# 206-IL
SCF	R&D	Cat# 255-SC
IL3	R&D	Cat# 203-IL
TGFβ	Peprotech	Cat# 100-21
IL-34	Peprotech	Cat# 200-34
M-CSF	Peprotech	Cat# 300-25
Fractalkine (CX3CL1)	Peprotech	Cat# 300-31
CD200	Novoprotein	Cat# C311
DAPI (4′,6-diamidino-2-phenylindole)	Invitrogen	Cat# D1306
Matrigel Matrix Growth Factor Reduced	Corning	Cat# 354230
mTeSR Plus	STEMCELL Technologies	Cat# 100-0276
Astrocyte Medium kit	ScienCell	Cat# 1801
Accutase	Millipore	Cat# SCR005
TrypLE Select Enzyme	Gibco	Cat# 15240062
Antibiotic-Antimycotic	Gibco	Cat# 15240096
N2 Supplement	Gibco	Cat# 17502048
B27 with Vitamin A	Gibco	Cat# 17504044
B27 without Vitamin A	Gibco	Cat# 12587010
Monothioglycerol (MTG)	Sigma	Cat# M1753
GlutaMax Supplement	Gibco	Cat# 35050061
Non-essential Amino Acids (NEAA)	Gibco	Cat# 11140050
Insulin-Transferrin-Selenium (ITS-G)	Gibco	Cat# 41400045
Insulin-Transferrin-Selenium-Ethanolamine (ITS-X)	Gibco	Cat# 51500056
Chemically-defined lipid concentrate	Gibco	Cat# 1190503
Beta-mercaptoethanol	Sigma	Cat# M6250
Brain-derived neurotrophic factor (BDNF)	R&D	Cat# 248-BD-025
Glia-derived neurotrophic factor (GDNF)	R&D	Cat# 212-GD-050
Dibutyryl cyclic AMP sodium salt (cAMP)	Sigma	Cat# D0627
L-Ascorbic acid	Sigma	Cat# A0278
Laminin Mouse Protein	Gibco	Cat# 23017015
KnockOut™ Serum Replacement	Gibco	Cat# 10828028
Dimethyl sulfoxide (DMSO)	Sigma	Cat# D8418

**Other**		

IMDM	Gibco	Cat# 12440053
F12	Gibco	Cat# 11765054
DMEM/F12 with GlutaMax	Gibco	Cat# 10565018
DMEM/F12 no phenol red	Gibco	Cat# 21041025
DMEM high glucose	Gibco	Cat#11965092
BrainPhys	STEMCELL Technologies	Cat# 05790
Nitrogen gas	Linde	Cat# NI T
Carbon dioxide gas	Linde	Cat# CD 50
EVOS Microscope	Invitrogen	Cat# AMF7000HCA
Countess 3 Automated Cell Counter	Invitrogen	Cat# AMQAX2000
Multi-gas incubator	Panasonic	Cat# P-MCO170AICUVL
BSL 2 safety cabinet	ESCO	Cat# AC2-6E8
Nalgene® SYSTEM 100™ Cryogenic Vials	Thermo Scientific	Cat# 66008-710

## Step-by-step method details

All the in-house generated hiPSC lines (TCW et al., 2022) and the commercially available KOLF2.1J hiPSC line have been authenticated for mycoplasma negative and normal karyotypes. All experiments are performed in a BSL2 safety cabinet.

### Differentiate hiPSCs to microglia (steps 1–8)

**Timing: 38 days**

The following steps outline the stages of hiPSC differentiation into HPCs (an intermediate cell type) and microglia (final). We describe here the generation of highly pure and expandable HPCs via hypoxia without additional sorting processes and the differentiation of homeostatic microglia that eventually mature by exposure to combinations of cytokines.

#### Day −1

1. Dissociate hiPSC into single cells:
  a. Aspirate the complete mTeSR Plus medium.
  b. Wash cells once with DPBS (1x) and aspirate.
  c. Add 1 mL of TrypLE or Accutase per 6-well and incubate at 37°C and 5% CO_2_ for 5 min.
    **CRITICAL:** It is not recommended to use Accutase unless cells have been continuously dissociated with Accutase.
  d. Dissociate hiPSC by gently tapping and pipetting. Collect cells in a 15 mL centrifuge tube.
  e. Wash and dissociate the remaining cells with 1 mL of complete mTeSR Plus medium and transfer to the 15 mL centrifuge tube.
  f. Centrifuge at 100 x g for 3 min.
  g. Resuspend the cell pellet in the complete mTeSR Plus medium supplemented with 10 μM Thiazovivin (Tzv).
  h. Reconstitute cell solution to 3 x 10^5^ cells/mL and seed 6 x 10^4^ cells/cm^2^ on a tissue-culture treated 6-well plate that is NOT coated with Matrigel.
  i. Incubate at 37°C and 5% CO_2_.
    **CRITICAL:** The seeding density is critical for initial differentiation.

#### Day 0

2. On the next day, change to HPC Day 0 media:
  a. Collect floating hiPSCs along with the supernatant into a 15mL tube.
  b. Centrifuge at 100 x g for 3 min.
  c. Aspirate the supernatant.
  d. Resuspend the cell pellet in pre-equilibrated Day 0 HPC differentiation medium (Table 6).
  e. Transfer cells back to the original 6-well plate with 2 mL medium/well.
  f. Incubate cells in a humidified multi-gaseous hypoxia incubator (5% O_2_ and 5% CO_2_) at 37°C for 2 days.

#### Day 2

3. Change to HPC Day 2 media:
  a. Collect the floating cells into a 15mL tube and centrifuge at 100 x g for 3 min.
  b. Aspirate the supernatant.
  c. Resuspend the cell pellet in the pre-equilibrated Day 2 HPC differentiation medium (Table 7).
  d. Transfer cells back to the original 6-well plate with 2 mL medium/well.
  e. Incubate cells in a humidified multi-gaseous hypoxia incubator (5% O_2_ and 5% CO_2_) at 37°C for 2 days.

#### Day 4

4. Change to HPC Day 4 media:
  a. Collect the floating cell clusters into a 15mL tube and centrifuge at 100 x g for 3 min.
  b. Aspirate the supernatant.
  c. Resuspend in the pre-equilibrated Day 4 HPC differentiation medium (Table 8).
  d. Transfer cells back to the original 6-well plate with 2 mL of medium per well.
  e. Incubate in normoxia (atmospheric oxygen level, 5% CO_2_) at 37°C for 6 days.
  f. Add 2 mL of fresh HPC differentiation medium per well every other day till Day 10.

**CRITICAL:** Avoid disturbing cells between Day 4 and Day 6 as HPCs do not firmly attach to the surface. Cells can easily be detached upon disturbance.

#### Day 10

5. Enrich the differentiated HPC and proceed to microglia differentiation:
  a. Filter the HPC through a 40 μm cell strainer.
  b. Centrifuge at 400 x g for 4 min.
  c. Aspirate the supernatant.
  d. Resuspend in 3 GF differentiation medium (Tables 9 and 10).
  e. Seed 2 x 10^4^ cells/cm^2^ in 2 mL of 3 GF differentiation medium per well on a Matrigel-coated 6-well plate.
  f. Incubate at 37°C and 5% CO_2_.
  g. Add 1 mL of 3 GF differentiation medium per well every other day.

**Figure 1:**
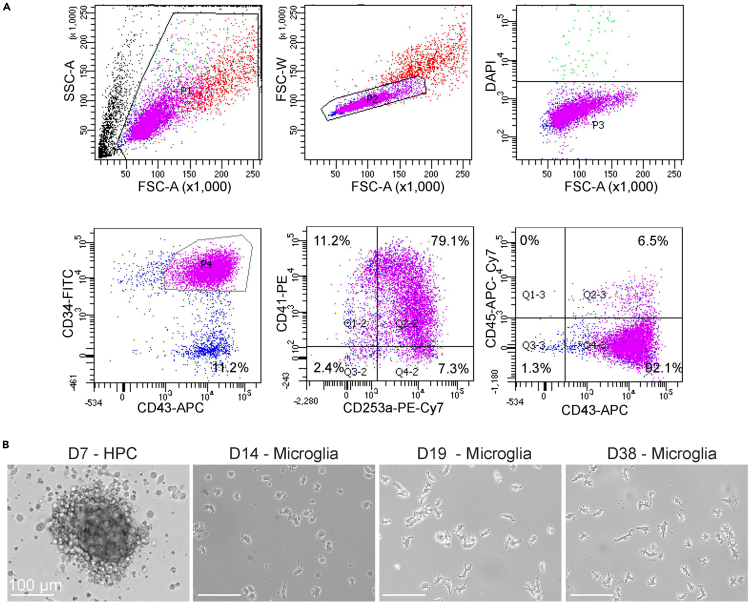
Characterization of the representative human HPCs (A) Flow cytometry on HPC markers, CD34, CD41, CD253a, CD45, and CD43. Cellular events were subjected to debris removal (top-left), doublet removal (top-middle), and stained with DAPI (top-right) to gate for the analysis of viable cells. A combination of surface markers was used to evaluate the HPC identity (bottom panel) with expression of 92.1% CD34+/CD43+/CD253a+/CD41+/CD45-cells. (B) Representative images of HPC differentiation from Day 7 (D7) HPC, Day 14 (D14), Day 19 (D19) microglia to Day 38 (D38) mature microglia. Scale bar: 100 μm.

***Note:*** The differentiated HPC has been characterized with HPC markers (CD34, CD253a, CD41, CD45, and CD43) by flow cytometry (Figure 1A). A high yield (92.1% purity) of HPCs is assured without immunological cell type-specific enrichment. Therefore, the sorting process for HPCs is removed after the validation.

#### Day 22

6. Completely refresh cells with 3 GF differentiation medium (Figure 1B):
  a. Collect floating cells in the supernatant and leave 1 mL of the conditioned cell medium per well.
  b. Filter the floating cells through a 40 μm cell strainer and collect in a 50 mL centrifuge tube.
  c. Centrifuge at 400 x g for 4 min.
  d. Aspirate the supernatant.
  e. Resuspend the cell pellet in a fresh 3 GF differentiation medium.
  f. Count and seed 2 x 10^4^ cells/cm^2^ in 2 mL of fresh 3GF differentiation medium on a fresh Matrigel-coated tissue-culture 6-well plate.
  g. Incubate at 37°C and 5% CO_2_.
  h. Add 1 mL of 3 GF differentiation medium per well every other day.

**CRITICAL:** Microglia might aggregate during differentiation. It is important to re-singularize the cells.

***Note:*** Day 22 is the estimated time to reach confluency in general (i.e., 7 x 10^5^ – 1 x 10^6^ cells per 6-well). Split cells at high confluency when needed.

#### Day 35

7. Change to 5 GF differentiation medium (Figure 1B):
  a. Collect all the floating cells into a 50 mL tube.
  b. Add 2 mL of 3 GF differentiation medium to the attached cells in each well.
  c. Filter the floating microglia through a 40 μm cell strainer.
  d. Centrifuge at 400 x g for 4 min.
  e. Aspirate the supernatant.
  f. Resuspend the cell pellet in 5 GF differentiation medium (Table 10).
  g. Replate approximately 7 x 10^4^ cells/cm^2^ in 2 mL of 5 GF differentiation medium on a fresh Matrigel-coated 6-well plate.
  h. Incubate at 37°C and 5% CO_2_.
  i. Add 1 mL of 5 GF differentiation medium per well every other day for another 3 days.
    ***Note:*** Microglia mature with reduced proliferation when exposed to 5 GF differentiation medium.

#### Day 38

**Figure 2:**
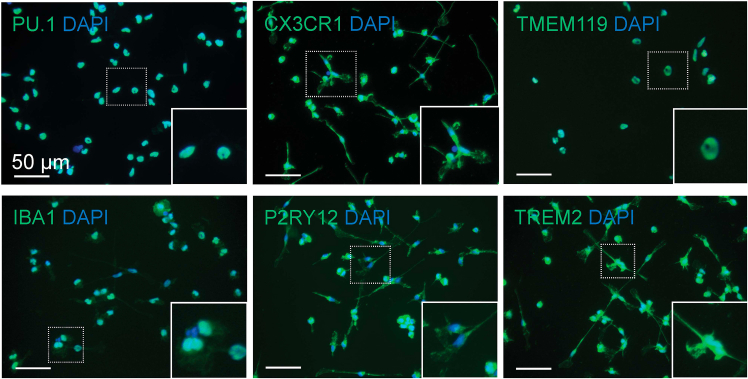
Representative immunofluorescent images of human microglia on Day 38 Top to bottom: PU.1, CX3CR1, TMEM119, IBA1, P2RY12, TREM2 positive human microglial markers showing microglial maturation. Scale bar: 50 μm.

8. Dissociate mature microglia by gentle pipetting for subsequent experiments. Perform immunocytochemistry to confirm the expression of mature microglia markers: CX3CR1, TMEM119, IBA1, P2RY12, PU.1, and TREM2 (Figure 2).
  ***Optional:*** Microglia cryopreservation:
  a. Collect the floating cells and attached cells by gentle pipetting into a 50 mL tube.
  b. Centrifuge at 400 x g for 4 min.
  c. Discard the supernatant.
  d. Resuspend cells in pre-chilled freezing medium (i.e., 10% DMSO in culture medium) or alternative appropriate cryopreservation medium.
  e. Aliquot cell suspension into 1 mL per cryogenic vial.
  f. Store in a cryopreservation container under −80°C.
    **CRITICAL:** Microglia do not survive well for freeze and thaw.

### Differentiate hiPSCs to astrocytes (steps 9–16)

**Timing: 42 days (14 days of NPC + 3 days of NPC expansion + 25 days of astrocyte differentiation)**

Here, we outline the procedures of hiPSC differentiation to NPCs (an intermediate cell type), followed by astrocytes. We describe the necessity of adopting MACS and co-expression of NPC identity markers (e.g., SOX2, PAX6, NESTIN) as a quality-control assessment to measure NPC purity to ensure the success and reproducibility of the differentiation.

#### Day −1

9. Dissociate 2 x 6-well of hiPSC at 80% confluency into single cells:
  a. Aspirate complete mTeSR Plus medium.
  b. Wash once with DPBS (1x) at room-temperature.
  c. Add 1 mL TrypLE or Accutase to each well and incubate at 37°C and 5% CO_2_ for 5 min.
  d. Dissociate hiPSCs by gentle tapping and pipetting.
  e. Transfer the dissociated cells to a 15 mL tube.
  f. Wash and collect the remaining cells with 1 mL of the complete mTeSR Plus medium in the same tube.
  g. Centrifuge at 100 x g for 3 min.
  h. Discard the supernatant.
  i. Resuspend cell pellet in the complete mTeSR Plus medium supplemented with 10 μM Tzv.
  j. Seed 5 x 10^4^ hiPSCs in 200 μL of mTeSR Plus per well in a low attachment round-bottomed 96-well plate or 8 x 10^5^ hiPSCs in 2 mL of the media in a low attachment 6-well plate.
  k. Collect cells at the bottom by centrifuging at 100 x g for 3 min.
  l. Incubate at 37°C and 5% CO_2_.

#### Day 0

**Figure 3:**
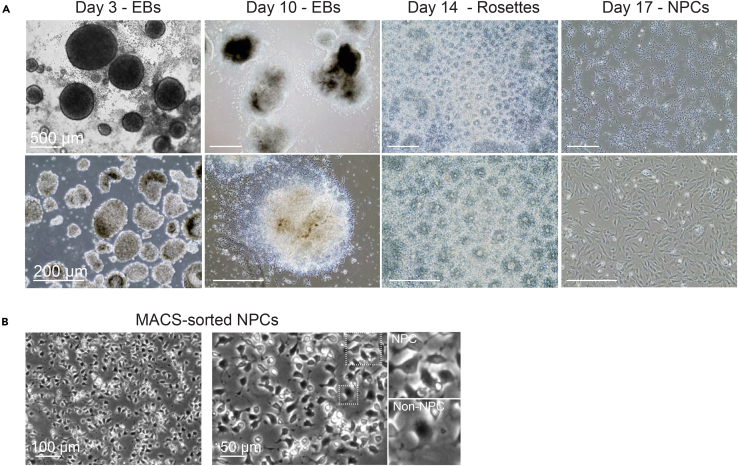
Representative bright-field images of the NPC differentiation process (A) Stages of EB differentiation (Day 3 on low attachment plates and Day 10 on Matrigel-coated plate), rosette formation (Day 14), and selected NPC (Day 17). Scale bar: 500 μm (top panel), 200 μm (bottom panel). (B) Morphological differences of NPCs and non-NPCs in bright-field images. Scale bar: 100 μm (left), 50 μm (right).
  c. Change the medium every other day.

10. Start EB differentiation from hiPSC:
  a. Carefully remove mTeSR Plus medium from each 96-well by using a multichannel aspirator.
  b. Refresh cells with 200 μL of the EB differentiation medium (Table 11) without disturbing the cells (Figure 3).

#### Day 7

11. Collect EBs and plate them on Matrigel-coated plates:
  a. Dislodge EBs from 96 wells by gently flushing with 100 μL of EB medium per well (Table 11) or collect EBs from a low-attachment-6-well plate to a 15 mL tube.
  b. Transfer the collected EBs to 3 x 6-wells in a Matrigel-coated 6-well plate and distribute evenly.
  c. Add 3 mL of the EB differentiation medium per well.
  d. Incubate at 37°C and 5% CO_2_.
  e. Refresh medium every other day.

#### Day 14

12. Rosette selection from the attached EBs:
  a. Aspirate medium and wash once with DPBS (1x) at room-temperature.
  b. Add 1 mL of the rosette selection reagent per well.
  c. Incubate at 37°C and 5% CO_2_ for 45 - 60 min maximum.
  d. Aspirate the rosette selection reagent.
  e. Add and expel 1 mL of NPC differentiation medium by directly pipetting with P1000 to the clumpy colony area to dislodge the rosettes.
  f. Transfer the dissociated rosettes to a 15 mL tube.
  g. Repeat steps of e) - f) until all rosettes are collected.
  h. Centrifuge at 300 x g for 4 min.
  i. Discard the supernatant.
  j. Gently dissociate large cell clumps by pipetting with 3mL of NPC medium (Table 12) with a 5mL serological pipette.
  k. Fill up the volume to a total 6 mL with another 3mL of NPC medium.
  l. Add 2 mL of the cell solution per well to 3 x wells on a Matrigel-coated 6-well plate.
  m. Incubate at 37°C and 5% CO_2_.
  n. Change medium every other day until cells reach confluency (Figure 3).

**CRITICAL:** The incubation time of the rosette selection agent and the frequency of expelling washes should be well-controlled to minimize the collection of contaminating non-neural progenitor cells. Check rosette dissociation under the microscope to determine the completion of extraction.

#### Day 17

**Figure 4:**
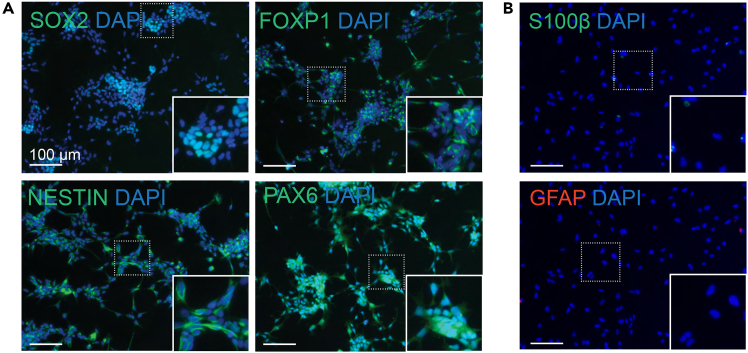
Validation of the NPC identity by immunostaining (A) Representative images of essential NPC markers: SOX2, FOXP1, NESTIN, and PAX6. Scale bar: 50 μm. (B) Negative staining of S100β and GFAP to show the absence of glial cell contamination. Scale bar: 50 μm.
  k. Passage NPCs at approximately 1:3 with Accutase when cells reach 100% confluency. Target seeding density is about 1 x 10^6^ cells per 6-well, although the optimal density may differ by cell line.

***Note:*** NPCs can be expanded up to 14 passages. Upon decreasing the purity of NPCs, cells can be re-sorted by repeating a) - i).

13. Purify and enrich NPC population by MACS:
  a. Dissociate NPCs by adding 1 mL of Accutase per well.
  b. Incubate at 37°C and 5% CO_2_ for 5 min.
  c. Gently pipette to dissociate into single cells, filter through a 40 μm cell strainer, and transfer to a 15 mL tube.
  d. Centrifuge at 400 x g for 4 min.
  e. Discard the supernatant.
  f. Follow manufacturer’s instructions to MACS NPCs by a negative selection to deplete CD271^+^ neural crest stem cells followed by a positive selection for CD133^+^ NPCs.
  g. To recover pure NPCs following MACS, seed at around 1 - 2 x 10^6^ cells per well on a Matrigel-coated 6-well plate and in 2 mL of the NPC medium supplemented with 10μM Tzv.
  h. On the next day, refresh cells with the NPC medium without Tzv.
  i. Refresh the medium every other day.
  j. For quality control, prepare a small portion of NPCs for immunohistochemistry by using the following markers: SOX2, FOXP1, NESTIN, and PAX6 (Figure 4A).

#### Day 18

14. Seed NPCs to differentiate astrocytes:
  a. Dissociate NPCs with Accutase and plate at 6.5 x 10^3^ cells/cm^2^ in astrocyte medium supplemented (Table 13) with Tzv on a Matrigel-coated plate.

**CRITICAL:** Initial NPC seeding density and single cell dissociation are critical, particularly during the first 25 days of differentiation in order to efficiently generate a homogenous population of astrocytes. NPC should not express GFAP or S100B (Figure 4B).

#### Day 19

15. Refresh with astrocyte medium:
  a. Refresh astrocyte medium and remove Tzv.
  b. Refresh the medium every other day.
  c. When the cells reach 80% confluency (approximately every 6–7 days), split to the initial seeding density (6.5 x 10^3^ cells/cm^2^) in astrocyte medium and cultured on Matrigel-coated plates.

**CRITICAL:** Seeding density is crucial for optimal astrocyte morphology and identity marker expression.

#### Day 42

16. Harvest mature astrocytes for experiments (Day 25 - 30 in astrocyte medium).

**Figure 5:**
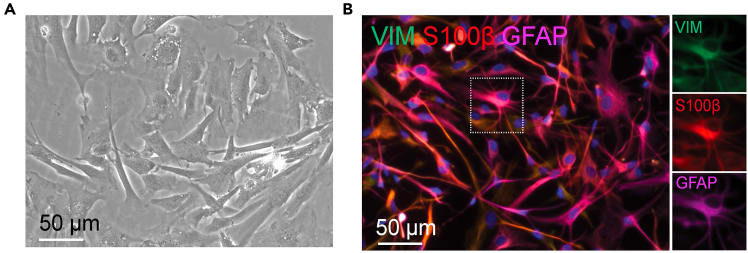
Representative immunofluorescent images of mature human astrocytes (A) A bright field image of Day 30 mature astrocytes. Scale bar: 50 μm. (B) Representative images of immunostaining of astrocytic markers: VIM, S100β, and GFAP. Scale bar: 50 μm.

***Note:*** From Day 25 and onwards, cells should express mature astrocyte markers including VIM, S100B or GFAP (Figure 5).

***Optional:*** After Day 25, split astrocytes 1:3 every week with Accutase. Astrocytes may be expanded up to 120 days in astrocyte media containing 2% (v/v) FBS or can survive and expand for up to 30 days without FBS.

**Figure 6:**
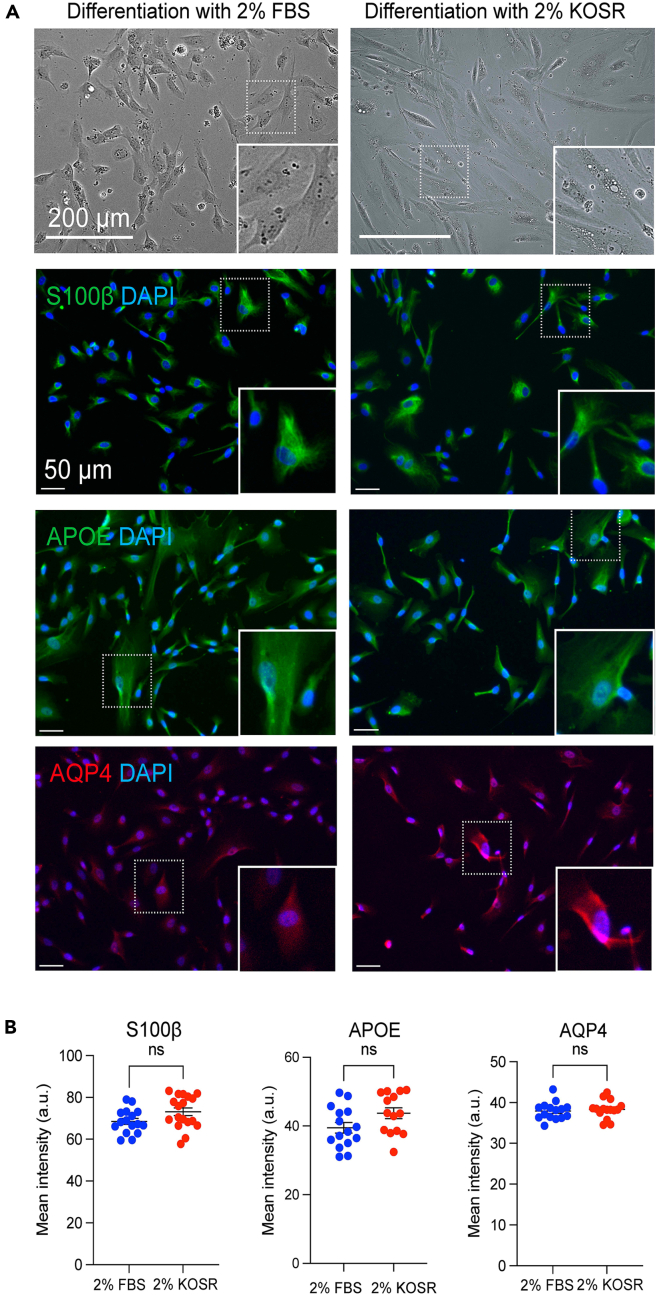
Representative immunofluorescent images of human astrocytes differentiated with serum vs. KnockOut Serum Replacement (KOSR) Human astrocytes were exposed to 2% KOSR vs. 2% FBS for 48 h following 25 days of differentiation. (A) Representative images. Top to bottom: bright field images, scale bar: 200 μm. S100β, APOE, and AQP4 staining with DAPI, scale bar: 50 μm. (B) Quantification of S100β, APOE, and AQP4 mean intensity in astrocytes. Dot plot: mean ± SEM. n = 3 independent experiments, 5 - 6 FOV per experiment. Two-tailed Student’s *t* test. ns: non-significant, ∗p < 0.05, ∗∗p < 0.01, ∗∗∗p < 0.001, ∗∗∗∗p < 0.0001.

***Optional:*** Astrocyte differentiation without serum: replace 2% (v/v) FBS with 2% KnockOut Serum Replacement (KOSR) in the astrocyte medium. Cells can be further differentiated for 48 h from Day 28 to Day 30. KOSR-treated astrocytes yield equivalent expressions of astrocytic markers, S100β, APOE, and AQP4 and similar morphology compared with FBS-treated astrocytes. (Figure 6).

**CRITICAL:** Astrocytes in extended culture maintenance beyond 120 days may have difficulty in enzymatic dissociation with Accutase.

### Differentiate NPCs to MCCs (steps 17–22)

**Timing: 42 days**

With the derived NPCs from above, we outline here the procedures for differentiating MCCs. The MCC culture contains a mixture of excitatory and inhibitory neurons and astrocytes, with glutamatergic neurons as the primary cell type.

#### Day 0

17. Seed MACS-sorted NPC and initiate the differentiation of MCC:
  a. Dissociate and singularize NPCs with Accutase.
  b. Seed at 4.2 x 10^4^ - 1 x 10^5^ cells/cm^2^ on a Matrigel-coated plate in the MCC differentiation medium (Table 14) supplemented with 10 μM Tzv.
  c. Incubate at 37°C and 5% CO_2_.

#### Day 1

18. Completely refresh cells with 2 mL of the MCC differentiation medium and remove Tzv.
19. Incubate at 37°C and 5% CO_2_.

#### Day 3

20. Perform gentle half-medium change with a 10 mL serological pipette.
21. Repeat every other day till Day 42.

#### Day 42

**Figure 7:**
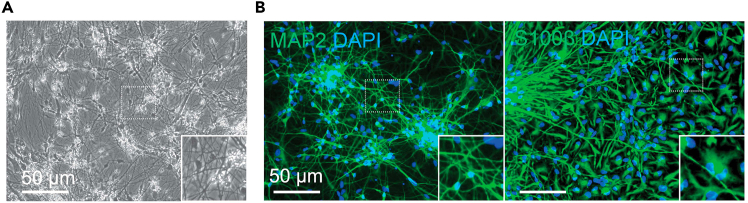
Representative images of hiPSC-derived MCC (A) A bright field image of Day 42 MCC. Scale bar: 50 μm. (B) Representative immunostaining images of neuronal marker MAP2 and DAPI (left) and astrocyte marker S100B and DAPI (right). Scale bar: 50 μm.

22. Harvest MCCs for characterization with neuronal (MAP2) and glial (S100β) markers (Figure 7).

**CRITICAL:** The glial proportion might increase at the end of MCC differentiation due to impure NPC with the presence of contaminant neural crest stem cells.

## Expected outcomes

### Microglia differentiation

The derived HPCs are subjected to a flow cytometry validation and should express a combination of MHC class II proteins CD34+/CD43+/CD253a+/CD41+/CD45- (Figure 1). Hypoxia facilitates the generation and self-renewal of the HPC niche by predisposing hypoxia-inducible factor (HIF) pathways to mediate hematopoiesis and lymphoid differentiation of HPCs.^5^^,^^6^ Moreover, bone morphogenetic protein 4 (BMP4) is added to regulate mesodermal-to-hematopoietic transition of HPCs and promote the formation of hematopoietic colonies *in vitro*; activin A, on the other hand, is used to inhibit early B lymphopoiesis.^7^^,^^8^ Morphogens and hypoxia are, therefore, combined to direct hiPSCs to a hematopoietic lineage in this protocol. During hypoxic differentiation, HPCs are expected to loosely attach to the non-coated tissue culture-treated surface. After conversion of hypoxia to normoxia, the cells are expected to be continuously generated from the attached mesodermal colonies and proliferate at an exponential scale. Approximately 2 - 4 x 10^6^ HPCs per 6-well are expected by Day 10 of differentiation. Filtration of cells through a 40 μm cell strainer is sufficient to acquire single-cell levels of HPC population and enhance cell recovery and survival (Figure 1), thereby circumventing the long and harsh cell sorting process that might cause potential cell damages or external contamination by using fluorescent activated cell sorting (FACS). Filtered HPCs are then plated for microglia differentiation on the Matrigel-coated plate. The derived microglia should express PU.1 for irreversible commitment to the erythro-myeloid cell lineage and essential microglial markers such as CX3CR1, TMEM119, IBA1, P2RY12, and TREM2 (Figure 2). The microglia during differentiation are expected to develop ramified protrusions for environmental surveillance and begin to attach to the surface of Matrigel-coated plate along the maturation process.

### Astrocyte differentiation

Astrocyte differentiation follows the successful generation of pure NPCs from hiPSCs. After dissociation of hiPSCs in suspension, they spontaneously assemble into EBs (Figure 3). Dual SMAD inhibition by TGF-β receptor inhibitor (SB431542) and BMP signaling antagonists (Noggin or LDN 193189) reduces the pluripotency of hiPSCs in EBs and promotes cell differentiation towards the trophoblast and neuroectodermal lineages.^9^ The self-assembled EBs start to display radial columnar arrangements within spheres from Day 3 of differentiation, representing a hallmark for neuroepithelial tissue development.^10^^,^^11^ Those neural rosette-like structures are expected to mature further when EBs integrate with the extracellular matrix for continuous neural rosette differentiation *in vitro* (Figure 3A). After rosette selection and replating, a mixture of NPCs and neural crest stem cells is collected, in which NPCs display a triangular morphology in a small size, whereas neural crest stem cells are in a flat-spread morphology (Figure 3B). The mature NPCs show robust expressions of NPC markers: SOX2, FOXP1, NESTIN, and PAX6 but negative expressions of other cell type markers: S100B and GFAP (Figure 4). During astrocyte differentiation, morphological transition is readily observed as early as Day 7, in which cells appear flattened and enlarged, develop long and bushy processes, and undergo mitotic activities. Expression of astrocytic markers such as Vimentin, S100B, and GFAP can be detected after 25 days of differentiation (Figure 5).

### Serum-free condition for astrocyte differentiation and cultures

While protocols for microglia and MCC have no serum in the cocktail, the astrocyte protocol still includes 2% serum. We previously demonstrated 2% serum exposure does not activate astrocytes by global transcriptomic profiling.^4^ However, the serum use in the medium could be an issue in a particular research area such as lipid or cellular metabolic studies. It is also possible to generate inconsistent experimental results due to batch-to-batch serum variations. However, simple removal of serum or nutrient starvation also activates cellular clearance mechanisms,^12^ limiting certain targeted research areas. Thus, properly removing serum in astrocyte cultures without hindering cellular homeostasis is a key for proper glial cell research. We demonstrated this process by replacing the 2% FBS serum to 2% Knock-Out Serum Replacement (KOSR) in the astrocyte medium. Astrocytes with short-term exposure (48 h) of KOSR following 25 days of differentiation behave equivalent to those differentiated with the 2% serum culture protocol presented in this paper. There were no significant differences in the expressions of astrocyte-specific pan markers and maturation markers and morphology (Figure 6). Depending on the target research area, either protocol is recommended.

### MCC differentiation

MCC differentiation from NPCs is mediated by the modulation of neural patterning pathways through morphogen and small molecule treatments to mimic *in vivo* neural development. NPCs are seeded at a low density to facilitate synaptic outgrowth and neural differentiation. Thin and arborizing neurites are expected to develop within 7 days, and electrophysiologically functional neurons emerge from Day 21 and become mature in Day 30–42.^13^ As the MCC matures over time, the complexity of neuronal networks evolves with radially extending synapses and increased neuritic branching and synaptic density.^14^ A few mitotic glial cells positive for S100B (Figure 7) are expected to emerge during the cortical differentiation possibly due to heterogeneous starting NPCs, which possess contaminant neural crest stem cells that differentiate glia or due to various response of individual cells to morphogens at the developmental stage. Although glial presence has been observed, excitatory glutamatergic neurons constitute a majority of the neuronal population in MCC, and neuronal markers such as MAP2 or TUJ1 should be readily detected by Day 42 of the differentiation (Figure 7).

## Quantification and statistical analysis

### Astrocyte marker quantification

Immunocytochemistry intensity was quantified using Image-J. The image was preprocessed with a Gaussian filter and subtracted background by adjusting the rolling ball radius. The updated image was then duplicated with one copy masked with an adjusted threshold. Regions of interests (ROI) were obtained by particle analysis and applied to the original image for mean intensity measurement. The summarized value of all ROIs was used for quantification and visualization. Statistical analysis was performed by adopting a two-tailed Student’s *t* test to compare the effect of KOSR vs. FBS in astrocyte differentiation, n = 3 independent experiments for each condition with 5 - 6 fields of view per experiment.

## Limitations

hiPSC studies have fostered therapeutic and translational breakthroughs in diseases that lack well-defined pathogenic mechanisms and drug responsiveness. The potential to generate diverse cell types expressing disease signatures and patient-specific genetics makes hiPSCs a novel model for mechanistic studies and pharmacologic discovery. However, several caveats need to be addressed.

First, hiPSC reprogramming removes the age-associated epigenetics that may contribute to the disease development.^15^ In addition, hiPSC-derived MCCs highly resemble human fetal tissues, implicating the limitation of hiPSCs to model aging-associated phenotypes in neurodegenerative diseases.^15^^,^^16^ Trans-differentiation from fibroblasts, i.e., direct conversion from fibroblasts to neurons by transgene inductions, has emerged as an alternative to preserve age-associated epigenetics, while the source fibroblasts are limited resources. Secondly, the proliferation capability and cell-type identity characteristics of the differentiated astrocytes gradually declines as the cells undergo extended cultures, which might implicate replicative senescence due to telomere shortening and DNA damage repair responses in cellular aging.^17^ Therefore, without genetic modification like introduction of human telomerase reverse transcriptase (hTERT)^18^ the differentiated astrocytes are impossible for unlimited expansion like immortalized cell lines. The optimal window for assay is recommended between Day 25 and Day 60 of the differentiation. Third, directed differentiation requires lengthy processes including proper quality control steps and is costly considering the consumption and cost of various morphogens (growth factors) throughout the protocols. Fourth, hiPSC clonal variability and individual genetic backgrounds need to be considered as attributes to potential phenotypic heterogeneity. The clonal variations within the same individual could be derived from the source of various somatic cell types, lineage-specific DNA methylation of somatic cells, or various iPSC reprogramming methods.^19^ Therefore, for the modeling perspectives, it emphasizes the necessity of multiple biological and clonal replicates. Despite the yields being variable among lines or clones, the presented protocol here has been tested more than 150+ times with 100% success rate.

Microglial activity is environment sensitive. Chemotactic nucleotides like ATP released from apoptotic cells act as a ‘Find-me’ signal, which interact with the P2Y receptor family on the cell surface and enhance the phagocyte recruitment and phagocytosis.^20^ Microglial activation not only promotes phagocytosis but also produces cytotoxic or proinflammatory cytokines, which damage healthy cells in culture. Furthermore, this apoptosis-induced phagocytosis has been found to underpin both rapid-transcriptomic and lasting-epigenetic alterations in microglia,^21^^,^^22^ which may induce phagocytic programming and long-term functional alteration of the innate immunity responding in response to repeated exogenous or endogenous stimuli.^23^ These inflammatory insults introduce variability of microglial population in a dish culture and potentially inducing the whole culture microglial activation, phagocytosing each other, leading to cell death. Therefore, it is critical to optimize microglial culture conditions to minimize cell death by maintaining a majority of microglia alive rather than dead, which could activate the ‘eat-me’ cascade.

Lastly, MCC differentiation is time-consuming. Assurance of the starting NPC quality is therefore important to generate quality cultures that represent the human brain cortex for disease modeling. Meanwhile, the derived culture presents a variety of neural cell types including a minor proportion of GABAergic and dopaminergic neurons in addition to a majority of glutamatergic neurons and glial cells. These minor portions of neuronal subclasses start to appear after 3 weeks of neuronal maturation and generate active AMPA and GABA fed-in synapses to activate or strengthen action potentials.^13^ Because MCCs inherently follow individual genetics and fetal brain developments, relative cell-type proportions slightly vary among individuals,^3^ which requires de-convolution of transcriptomic analyses to correct for effects of different cell proportions. Meanwhile, the quality of early passage NPC differentiation and NPC sorting as quality control and maintenance processes determine the high-quality neuronal cultures in MCCs.

## Troubleshooting

### Problem 1

Microglia aggregate, forming floating clusters, and do not attach to the Matrigel-coated plate during differentiation (related to Step 6).

### Potential solution

Under suboptimal culture conditions such as too high cell confluency (e.g., > 1.5 x 10^5^ cells/cm^2^), the derived microglia may detach and aggregate. We recommend dissociating these microglia aggregates into single cells by gentle pipetting and removing the cell clumps by filtering through a 40 μm cell strainer. Replate the singularized cells at 2 x 10^4^ cells/cm^2^ on a Matrigel-coated plate and incubate cells at 5% CO_2_, room temperature for 10 - 20 min. The seeding density requires optimization per individual cell line. Collect floating cells in a 15 mL centrifuge tube and refresh the attached cells with the microglia differentiation medium (Tables 8 and 9). At the same time, pellet the floating cells by centrifugation at 400 x g for 4 min. Discard the supernatant and resuspend the cell pellet in 1 mL of the microglia differentiation medium. Measure cell survivability with Trypan blue. If > 80% of cells are alive, plate the cells back on a fresh Matrigel-coated plate in an appropriate amount of medium. If cell viability is low < 50%, discard the pellet.

### Problem 2

The differentiated microglia show poor survivability after cryopreservation (related to Step 8).

### Potential solution

We recommend using poly-D-lysine coating to facilitate the attachment of microglia after thawing. To recover microglia from cryopreservation, thaw cells in a water bath. Add 1ml of microglia differentiation medium per cryogenic vial when cells are completely thawed. Do not pipette to physically break the frozen ice block of cells. Transfer total 2 mL of the cell solution to a 15 mL tube. Rinse the cryogenic vial with additional 1 mL of the microglia differentiation medium and collect in the same centrifuge tube. Centrifuge at 400 x g for 4 min. Aspirate the supernatant and resuspend cells with the microglia medium. Gently resuspend and seed 5 - 7 x 10^4^ cells/cm^2^ on a poly-D-lysine-coated plate and incubate at room temperature and 5% CO_2_ for 10 - 20 min. Aspirate the supernatant and add 2 mL of microglia differentiation medium supplemented with 10μM Tzv. Incubate at 37°C and 5% CO_2_. Remove Tzv on the next day. Add the microglia differentiation medium every other day and remove floating cell clumps as described above until cells recover from cryopreservation.

### Problem 3

Astrocytes are not detaching from the plate during cell collection by Accutase for passaging (related to Step 16).

### Potential solution

Astrocytes become more difficult to detach from the Matrigel-coated plate as they approach 60 days of the culture. When dissociating those astrocytes with Accutase for passaging, confirm cell detachment first under a brightfield microscope. If necessary, extend the incubation time with Accutase in 37°C (1 h max) to ensure cells are detached and in suspension. When retrieving cells, pipet the suspension solution to dislodge any cells that may still be adhered on the plate. However, if cells are still adherent on the plate after multiple rounds of pipetting, it is recommended to be discarded due to potential transformation and loss of astrocyte characteristics.

### Problem 4

Astrocytes having thin, elongated morphology is not suitable for immunocytochemistry and microscopy of cytoplasmic markers (related to Step 16).

### Potential solution

High density seeding renders astrocytes in a tailed, thin, and elongated morphology. Human astrocytes cultured at a lower density display enlarged cell bodies with an ample area for imaging of the cytoplasmic markers. Accordingly, we recommend seeding astrocytes at a density of 2.5 x 10^4^ in a well of the 24-well plate for immunocytochemistry. Individual cell lines may differ in cell body sizes, so it is necessary to adjust the seeding density for different cell lines.

### Problem 5

Too many mitotic and glial cells produced in the MCCs (related to Step 22).

### Potential solution

High glial presence might result from the contamination of neural crest stem cells during NPC differentiation. We suggest repeating the MACS sorting process to enrich homogeneous NPC population before MCC differentiation, thereby reducing the glial contamination. Additionally, bulk analyses of the entire culture are less favored if an overwhelming amount of mitotic glial cells is observed; single-cell level analyses on the targeted cell types are rather preferred.

## Resource availability

### Lead contact

Further information and requests for resources and reagents should be directed to and will be fulfilled by the lead contact, Julia TCW (juliatcw@bu.edu).

### Technical contact

Technical questions on executing this protocol should be directed to and will be answered by the technical contact, Julia TCW (juliatcw@bu.edu).

### Materials availability

This study did not generate new unique reagents.

### Data and code availability

The published article includes all datasets generated or analyzed during this study.

## Acknowledgments

Protocol development was supported by National Institutes of Health K01AG062683. We acknowledge additional support from the National Institutes of Health (R01AG082362 and R01AG083941), the Carol and Gene Ludwig Family Foundation, the Edward N. & Della L. Thome Memorial Foundation Awards Program in AD Research, Health Resources in Action, and the Keren Toffler Charitable Trust. The graphical abstract was adopted from BioRender.com.

## Author contributions

J.TCW. developed the protocols and secured funding. J.TCW. and L.Q. wrote the draft and generated figures. J.TCW., L.Q. and J.-G.R. revised the figures. All authors read and revised the manuscript.

## Declaration of interests

The author J.TCW. has a provisional patent application.
